# Coronary inflammation based on pericoronary adipose tissue attenuation in type 2 diabetic mellitus: effect of diabetes management

**DOI:** 10.1186/s12933-024-02199-x

**Published:** 2024-03-29

**Authors:** Yuankang Liu, Lisong Dai, Yue Dong, Cong Ma, Panpan Cheng, Cuiping Jiang, Hongli Liao, Ying Li, Xiang Wang, Xiangyang Xu

**Affiliations:** 1 Department of Radiology, Liyuan Hospital, Tongji Medical College, Huazhong University of Science and Technology, 39 Yanhu Avenue, Wuchang District, Wuhan, 430077 China; 2 Department of Diagnostic and Interventional Radiology, Shanghai Sixth People’s Hospital, Shanghai Jiao Tong University School of Medicine, No. 600, Yishan Road, Xuhui District, Shanghai, Shanghai, 200233 China; 3 Department of Surgery, Liyuan Hospital, Tongji Medical College, Huazhong University of Science and Technology, 39 Yanhu Avenue, Wuchang District, Wuhan, 430077 China; 4 Department of Radiology, The Central Hospital of Wuhan, Tongji Medical College, Huazhong University of Science and Technology, Shengli Road No. 26, Jiangan District, Wuhan, 430014 China

**Keywords:** Type 2 diabetes mellitus, Coronary computed tomography angiography, Perivascular coronary inflammation, Diabetes management, Pericoronary adipose tissue

## Abstract

**Background:**

Coronary inflammation plays crucial role in type 2 diabetes mellitus (T2DM) induced cardiovascular complications. Both glucose-lowering drug interventions (GLDIS) and glycemic control (GC) status potentially correlate coronary inflammation, as indicated by changes in pericoronary adipose tissue (PCAT) attenuation, and thus influence cardiovascular risk. This study evaluated the impact of GLDIS and GC status on PCAT attenuation in T2DM patients.

**Methods:**

This retrospective study collected clinical data and coronary computed tomography angiography (CCTA) images of 1,342 patients, including 547 T2DM patients and 795 non-T2DM patients in two tertiary hospitals. T2DM patients were subgroup based on two criteria: (1) GC status: well: HbA1c < 7%, moderate: 7 ≤ HbA1c ≤ 9%, and poor: HbA1c > 9%; (2) GLDIS and non-GLDIS. PCAT attenuations of the left anterior descending artery (LAD-PCAT), left circumflex artery (LCX-PCAT), and right coronary artery (RCA-PCAT) were measured. Propensity matching (PSM) was used to cross compare PCAT attenuation of non-T2DM and all subgroups of T2DM patients. Linear regressions were conducted to evaluate the impact of GC status and GLDIS on PCAT attenuation in T2DM patients.

**Results:**

Significant differences were observed in RCA-PCAT and LCX-PCAT between poor GC-T2DM and non-T2DM patients (LCX: − 68.75 ± 7.59 HU vs. – 71.93 ± 7.25 HU, *p* = 0.008; RCA: − 74.37 ± 8.44 HU vs. − 77.2 ± 7.42 HU, *p* = 0.026). Higher PCAT attenuation was observed in LAD-PCAT, LCX-PCAT, and RCA-PCAT in non-GLDIS T2DM patients compared with GLDIS T2DM patients (LAD: − 78.11 ± 8.01 HU vs. − 75.04 ± 8.26 HU, *p* = 0.022; LCX: − 71.10 ± 8.13 HU vs. − 68.31 ± 7.90 HU, *p* = 0.037; RCA: − 78.17 ± 8.64 HU vs. − 73.35 ± 9.32 HU, *p* = 0.001). In the linear regression, other than sex and duration of diabetes, both metformin and acarbose were found to be significantly associated with lower LAD-PCAT (metformin: *β* coefficient = − 2.476, *p*=0.021; acarbose: *β* coefficient = − 1.841, *p* = 0.031).

**Conclusion:**

Inadequate diabetes management, including poor GC and lack of GLDIS, may be associated with increased coronary artery inflammation in T2DM patients, as indicated by PCAT attenuation on CCTA, leading to increased cardiovascular risk. This finding could help healthcare providers identify T2DM patients with increased cardiovascular risk, develop improved cardiovascular management programs, and reduce subsequent cardiovascular related mortality.

**Supplementary Information:**

The online version contains supplementary material available at 10.1186/s12933-024-02199-x.

## Background

Diabetes mellitus is a metabolic chronic inflammatory disease associated with increased cardiovascular risk [1–3]. Against the backdrop of a sharp rise in the number of diabetes patients globally, type 2 diabetes mellitus (T2DM) patients, accounting for 90% of global diabetes cases, have become the primary group for cardiovascular disease-related deaths [4–6]. T2DM patients face a higher risk of cardiovascular complications due to exacerbated vascular inflammation, which leads to the remodeling of blood vessel structure and function, as well as reduced vascular elasticity and efficiency [7–9].

Pericoronary adipose tissue (PCAT) engages in a bi-directional signaling interaction with the arterial wall [10], not only secreting inflammatory cytokines towards the vessel wall but also responding to signals from it [11, 12]. This dynamic crosstalk is crucial for regulating vascular homeostasis under normal physiological conditions [13]. PCAT adipocytes constrict due to inflammatory factor secretion [14, 15]. This ‘cachexia’ effect on adipocytes near the inflamed arterial wall leads to lipid-poor adipocytes having increased water content in the proximal-to-distal direction. CCTA accordingly exhibits a CT value gradient - as PCAT nears the inflamed coronary wall, CT values rise [16]. The complex interactions between PCAT and the arterial walls play a critical role in maintaining vascular health. CCTA attenuation, as an imaging biomarker reflecting coronary inflammation, can assist in evaluating the status of vascular inflammation and cardiovascular disease risk [17].

A recent study found that diabetic patients exhibited significantly higher PCAT attenuation around the right coronary artery (RCA) compared with non-diabetic patients, irrespective of stenotic severity and plaque vulnerability [18]. potentially due to inflammation around blood vessels triggered by high blood sugar levels. Case studies have documented a consistent reduction in PCAT attenuation among T2DM following treatment with the antidiabetic drug somatostatin, hinting at GLDIS’s potential in mitigating inflammation of the coronary arteries [19]. Elevated PCAT attenuation is linked to a heightened risk of cardiovascular incidents [20, 21]. Considering this association, this study aimed to evaluate the effects of diabetes management strategies, especially GLDIS and the state of GC, on PCAT attenuation, aiming to contribute to the cardiovascular risk management for T2DM patients.

## Methods

### Study population

**Fig. 1:**
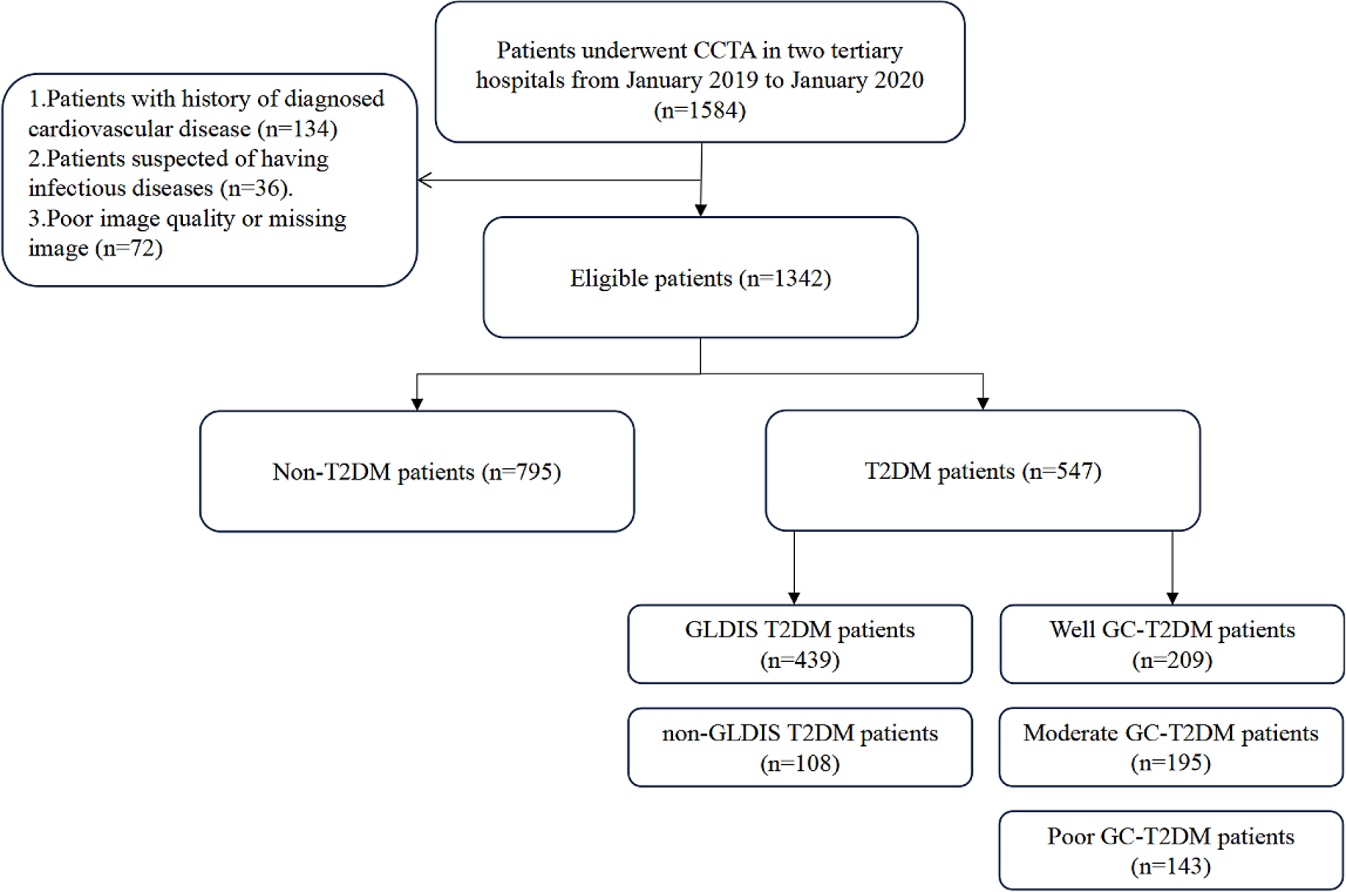
Flowchart showing the study design. *CCTA* coronary computed tomography angiography, *T2DM* type 2 diabetes mellitus, *GLDIS* glucose-lowering drug interventions

In this cross-sectional study, we retrospectively recruited consecutive T2DM patients and non-T2DM patients who underwent CCTA in two tertiary hospitals from January 2019 to January 2020. We gathered data on the clinical features, coronary CTA findings, and patient outcomes. The study protocol was approved by the Ethics Review Committee of Wuhan Central Hospital and the Ethics Review Committee of Liyuan Hospital (Wuhan Central Hospital: WHZXKYL 2023 − 168; Liyuan Hospital: [2023] IEC RYJ010), respectively. Finally, we included 1342 patients from Wuhan Central Hospital and Liyuan hospital. (Fig. 1 ).

### Data collection

From January 2019 to January 2020, patients who underwent CCTA and met the following inclusion criteria were enrolled: From January 2019 to January 2020, patients who underwent CCTA in two tertiary hospitals and did not meet the following exclusion criteria were included in the study : Exclusion criteria: (1) history of diagnosed cardiovascular disease (CVD), including myocardial infarction, ischemic heart disease surgery (coronary artery bypass grafting, percutaneous transluminal coronary angioplasty), or stroke. (2) suspected infectious diseases; and (3) poor image quality. The definition of T2DM includes the following criteria: (1) previous history of diabetes; (2) fasting blood glucose ≥ 7.0 mmol/L; (3) glycated blood glucose protein ≥ 6.5%; (4) receiving hypoglycemic therapy [22]. Based on HbA1c levels, T2DM patients are divided into three groups: well GC group: HbA1c < 7%, moderate GC group: 7 ≤ HbA1c ≤ 9%, and poor GC group: HbA1c > 9% [23]. GLDIS is defined as the use of glucose-lowering drug therapy, which includes the administration of one or more oral or injectable glucose-lowering drugs and maintaining this treatment for a period of 3 months or longer. Table 1 showed the results of how the groups were categorized and matched based on propensity scores.

**Table 1:** Propensity scores matching results

	T2DM patients					T2DM patients				GLDIS T2DM patients
	Well GC status	Moderate GC status	Poor GC status			GLDIS T2DM patients	Non-GLDIS T2DM patients			
Non-T2DM patients	163	136	98		Non-T2DM patients	271	90		Non-GLDIS T2DM patients	92

*T2DM* type 2 diabetes mellitus, *GC* glycemic control, *GLDIS* glucose-lowering drug interventions

A total of three radiologists jointly collected the baseline features of the patients from clinical inpatient records, such as age, sex, body mass index (BMI), laboratory test data, previous medication use, and CAD risk factors. Hypertension was defined as systolic blood pressure > 140 mmHg and/or diastolic blood pressure > 90 mmHg and/or use of antihypertensive medication [24]. According to the guidelines, dyslipidemia was defined as one or more of the following: total cholesterol > 6.2 mmol/L, low-density lipoprotein (LDL) cholesterol > 4.1 mmol/L, high-density lipoprotein (HDL) cholesterol < 1.0 mmol/L, serum triglycerides > 2.3 mmol/L, or diagnosis/treatment of dyslipidemia [25]. Smoking status is defined as current smoking or non-smoking. Data System (CAD-RADS) grade 3 or above were considered to have significant stenosis.

### CCTA acquisition

All patients underwent CCTA using a dual-source CT scanner (SOMATOM Definition Flash, Siemens Medical Solutions, Erlangen, Germany) or a 128-slice wide detector CT scanner (Revolution HD, GE Healthcare, USA). Patients’ heart rates were controlled to maintain approximately 70 beats per minute, and oral metoprolol was routinely recommended. CTA image acquisition was performed using prospective ECG-triggered Tube voltage (100–140 kV) and tube current was automatically adjusted based on the patient’s body size using the automatic exposure control system on the scanner.

### PCAT inflammation analysis

**Fig. 2:**
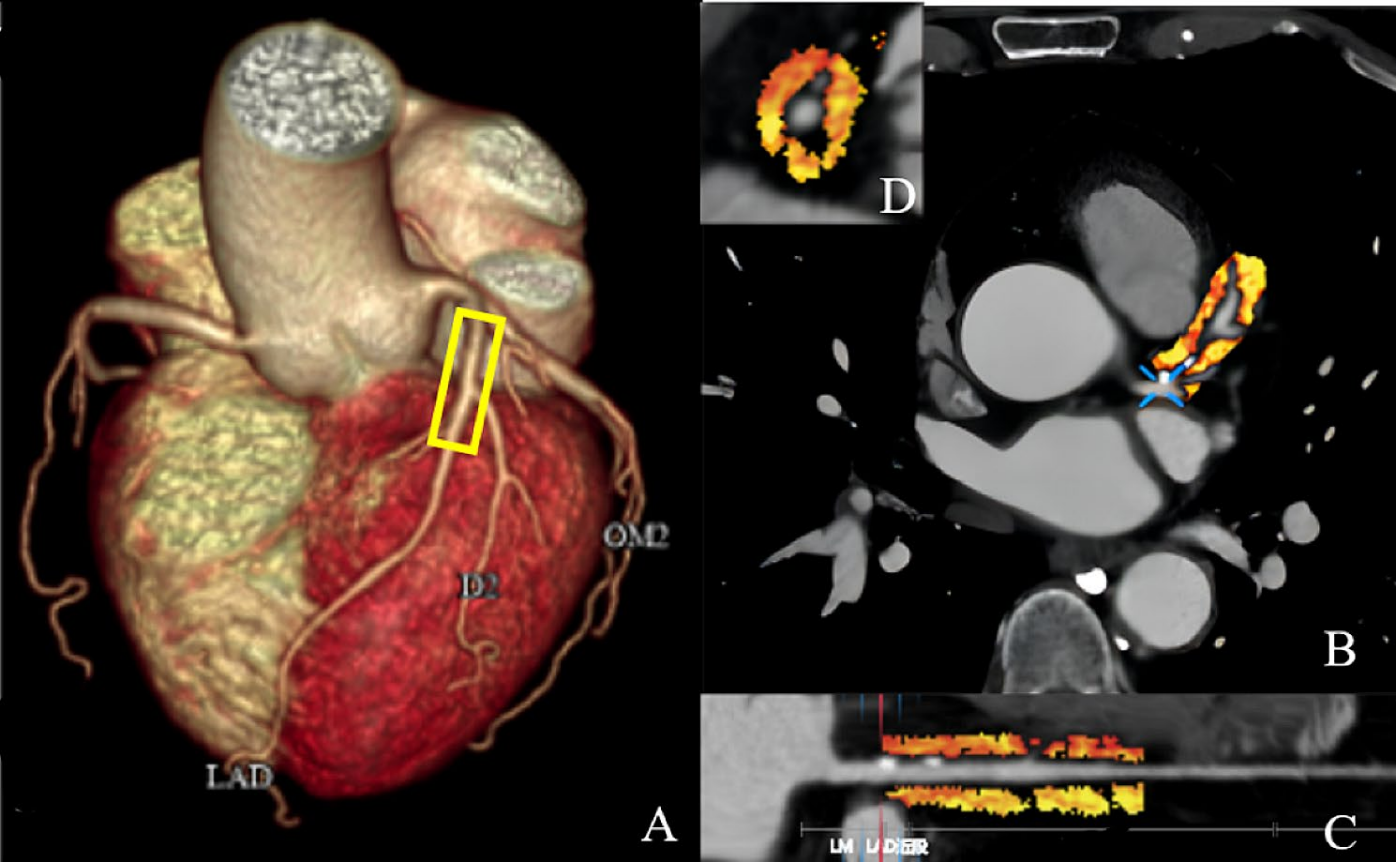
Representative case of LAD-PCAT attenuation measured by CCTA. **A** Three-dimensional reconstruction of the heart ; **B** PCAT attenuation between − 190 and – 30 HU in the cross-sectional view ; **C** The segment of the proximal coronary artery in a straightened view ; **D** Around the proximal 40 mm of the left anterior descending artery. *LAD* left anterior descending artery, *CCTA* coronary computed tomography angiography, *PCAT* Pericoronary adipose tissue

Using professional FAI analysis software (coronary artery FAI analysis, version 1.0.4, Shukun technology [26]), the three main vessels of the coronary artery—LAD, LCX, and RCA—were tracked. To avoid the influence of aortic wall, the opening of right coronary artery 10–50 mm and the proximal end of left anterior descending branch and left circumflex branch 40 mm were measured and measured. PCAT attenuation was obtained by automatically calculating and recording the weighted average CT attenuation of adipose tissue (attenuation coefficient between − 190 and − 30 HU) within the radial distance of the outer wall of the blood vessel equidistant from the average diameter of the blood vessel [17, 27]. Figure 2 provides an example of these parameters.

### Analysis of sample size

As there is no publicly available data on PCAT attenuation in GLDIS T2DM patients and non-GLDIS T2DM patients, the sample size was determined based on our own preliminary data. Prior to this study, we retrospectively analyzed the PCAT attenuation of 102 T2DM patients, who were not part of this study. The GLDIS T2DM patients (*n* = 87): RCA − 74.79 ± 7.34, non-GLDIS T2DM patients (*n* = 25): RCA − 78.74 ± 8.17. According to the findings, a sample size of 161 GLDIS T2DM patients and 47 non-GLDIS T2DM patients had to achieve a 90% efficacy level and observe significant differences at a unilateral significance level of 0.05 between GLDIS T2DM patients and non-GLDIS T2DM patients. Ultimately, the study included 439 GLDIS T2DM patients and 108 non-GLDIS T2DM patients, ensuring > 90% efficacy level. The sample size calculation was performed using the statistical software (PASS 2021 version 21.0.3,)

### Statistical analysis

Quantitative variables with a normal distribution were expressed as means ± standard deviations (SD), while median and interquartile range (IQR) were used for variables that were not normally distributed. Categorical variables were reported as counts (%) and compared using either the Chi-square test or Fisher exact test, depending on the size of the data cell. Student’s t-test was used for normally distributed data, and the Mann-Whitney U test was used for data that was not normally distributed. The assumption of a normal distribution was checked using the one-sample Kolmogorov-Smirnov test. One-way ANOVA with Bonferroni post-hoc test was used to compare continuous variables among multiple groups; the chi-squared test was used for comparison of categorical variables. To minimize the impact of baseline characteristic differences between the two groups, propensity score matching (PSM) was employed. A one-to-one nearest neighbor matching algorithm was used to assess the propensity score of each patient. A logistic regression model was used with T2DM as the dependent variable and age, gender, BMI, cardiovascular risk factors (dyslipidemia, hypertension, smoking), tube voltage and statin use as independent variables. Considering previous studies indicating the effect of statins on coronary inflammation, statins were also included in the matching factors [28].

Multivariate stepwise linear regression was conducted to examine the association between cardiovascular risk factors (age, gender, body mass index, dyslipidemia, hypertension, and smoking), glucose-lowering drugs, antihypertensive drugs, statin use, duration of diabetes, and tube voltage as independent variables, and LAD-PCAT, LCX-PCAT, and RCA-PCAT as dependent variables. Statistical significance was assessed using a two-tailed *p*-value of less than 0.05. All statistical analyses were conducted using SPSS Statistical Software version 25.0 and GraphPad Prism 7.0.

## Results

### Baseline characteristics of study groups

In Table 2, detailed demographic data are presented. A total of 1342 patients were included (mean age: 62.43 ± 10.58 years, including 662 males). At baseline, T2DM patients (*n* = 547) had a higher BMI (*p* = 0.019), a higher prevalence of hypertension (*p* < 0.001), dyslipidemia (*p* = 0.003), statin use (*p* < 0.001), and significant differences in all lipid profile measures. There was no significant difference in PCAT attenuation between T2DM and non-T2DM patients in the three coronary arteries (LAD: *p* = 0.238, LCX: *p* = 0.854, RCA: *p* = 0.671).

**Table 2:** Baseline characteristics

Variables		All	T2DM patients	Non-T2DM patients	*P*
*n*		1342	547	795	
Baseline characteristic					
	Age (years)	62.43 ± 10.58	62.35 ± 9.77	62.49 ± 10.71	0.829
	Male sex, n (%)	662 (49.3)	281 (51.3)	381 (47.9)	0.215
	Body mass index (kg/m2)	23.92 (22.13, 26.09)	24.22 (22.58,26.29)	23.73 (22.03,26.01)	0.019
	Smoking, n (%)	285 (21.2)	138 (25.2)	147 (18.5)	0.003
	Hypertension, n (%)	725 (54)	353 (64.4)	372 (46.9)	< 0.001
	Dyslipidemia, n (%)	632 (47.1)	352 (64.4)	280 (35.2)	< 0.001
Medications treatment					
	Statin, n (%)	666 (49.6)	345 (63.0)	321 (40.4)	< 0.001
	Antihypertensive drugs	435 (32.2)	223 (40.8)	212 (26.7)	< 0.001
Laboratory findings					
	Fast glucose (mmol/L)	5.85 (5.07,7.63)	7.84 (6.35,10.48)	5.15 (4.75,5.64)	< 0.001
	HbA1c (%)	5.95 (5.60,7.90)	7.7 (6.60,9.20)	5.5 (5.30,5.80)	< 0.001
	HDL-cholesterol (mmol/L)	1.22 (1.04,1.48)	1.13 (0.97,1.37)	1.28 (1.08,1.54)	< 0.001
	LDL-cholesterol (mmol/L)	2.81 (2.24,3.54)	2.62 (2.07,3.34)	3.00 ± 0.84	< 0.001
	Total cholesterol (mmol/L)	4.72 ± 1.21	4.58 ± 1.13	4.81 ± 1.22	0.001
	Triglyceride (mmol/L)	1.39 (0.93,2.11)	1.55 (0.99,2.39)	1.31 (0.91,2)	0.001
CCTA findings					
	Tube voltage of CT acquisition				0.001
	100 kVp, n (%)	773 (57.6)	297 (54.2)	475 (59.7)	
	120 kVp, n (%)	539 (40.2)	229 (41.8)	310 (39.4)	
	140 kVp, n (%)	31 (2.1)	21 (3.8)	10 (1.3)	
	DS	318 (23.7)	192 (35.1)	126 (15.8)	< 0.001
	LAD-PCAT (HU)	− 77.25 ± 8.05	− 77.64 ± 8.27	− 76.98 ± 7.93	0.238
	LCX-PCAT (HU)	− 71.30 ± 7.52	− 71.40 ± 8.04	− 71.23 ± 7.29	0.854
	RCA-PCAT (HU)	− 76.98 ± 8.24	− 76.84 ± 8.80	− 77.08 ± 7.42	0.671

Data are presented as means ± standard deviations or the median (25th and 75th percentile), with the interquartile range in parentheses or number (%)

*T2DM* type 2 diabetes mellitus, *HbA1c* glycated hemoglobin, *HDL* high-density lipoprotein, *LDL* low-density lipoprotein, *DS* diameter stenosis, *PCAT* Pericoronary adipose tissue, *LAD* left anterior descending artery, *LCX* left circumflex artery, RCA right coronary artery

### Comparison of clinical characteristics and CT parameters between non-T2DM and T2DM patients with different GC status

**Fig. 3:**
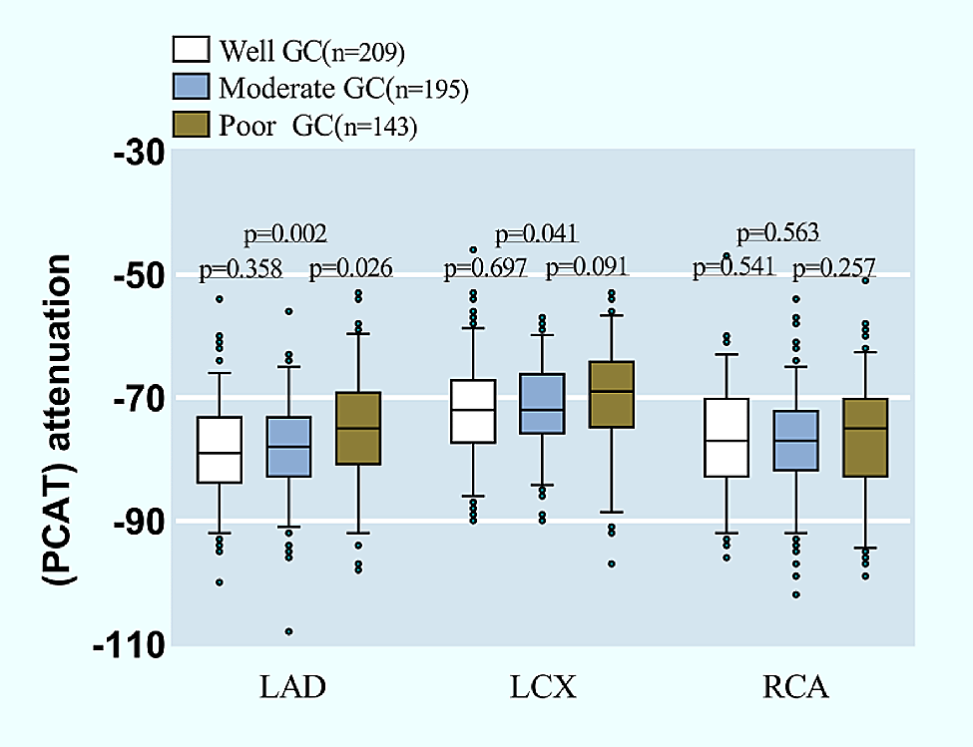
The pairwise comparisons of PCAT attenuation among T2DM patients with different GC statuses in three main coronary arteries. *PCAT* Pericoronary adipose tissue, *GC* glycemic control, *LAD* left anterior descending artery, *LCX* left circumflex artery, *RCA* right coronary artery

After adjusting for age, gender, BMI, cardiovascular risk factors (dyslipidemia, hypertension, smoking), tube voltage, CAD-RADS grade, and statin use, we observed a significant difference in RCA-PCAT between poor GC-T2DM patients and non-T2DM patients (− 74.37 ± 8.44 vs. − 77.2 ± 7.42, *p* = 0.026) (Table 3). Additionally, PCAT attenuation differences between well GC-T2DM patients or moderate GC-T2DM patients and non-T2DM patients were not significant. (Table 3) The pairwise comparisons of baseline PCAT attenuation around LAD, LCX, and RCA among T2DM patients with different GC statuses were shown in Fig. 3.

**Table 3:** Comparison between non-T2DM patients and T2DM patients with different GC status after matching

Variables	Non-T2DM vs. Poor GC-T2DM			Non-T2DM vs. Moderate GC-T2DM			Non-T2DM vs. Well GC-T2DM		
	Non-T2DM	Poor GC-T2DM	*P*	Non-T2DM	Moderate GC-T2DM	*P*	Non-T2DM	Well GC-T2DM	*P*
*n*	98	98		136	136		163	163	
Age (years)	60.13 ± 12.26	65.67 ± 9.32	0.001	62 (56.00, 67.00)	68 (57.00, 74.00)	0.001	64.44 ± 10.71	67.57 ± 11.23	0.010
Male sex, n (%)	47 (48.0)	52 (53.1)	0.475	63 (46.3)	75 (55.1)	0.146	69 (42.3)	94 (57.7)	0.577
Body mass index (kg/m2)	23.75 (22.14, 26.32)	24.69 (22.44, 26.71)	0.180	24.28 (22.79, 26.03)	24.68 (22.57, 26.48)	0.856	24.31 (22.04, 26.30)	24.44 (22.49, 26.57)	0.495
Smoking, n (%)	25 (25.5)	22 (22.4)	0.616	29 (21.3)	31 (22.8)	0.770	36 (22.1)	34 (20.9)	0.787
Hypertension, n (%)	58 (59.2)	64 (65.3)	0.377	84 (61.8)	87 (64.0)	0.707	108 (66.3)	96 (58.9)	0.170
Dyslipidemia, n (%)	57 (58.2)	52 (53.1)	0.472	65 (47.8)	58 (42.6)	0.394	60 (36.8)	61 (37.4)	0.909
Statin, n (%)	61 (62.2)	73 (74.5)	0.065	80 (58.8)	102 (75.0)	0.005	95 (58.3)	119 (73.0)	0.005
Tube voltage of CT acquisition			0.883			0.848			0.663
100 kVp, n (%)	52 (53.1)	54 (55.1)		67 (49.3)	71 (52.2)		85 (52.1)	93 (57.1)	
120 kVp, n (%)	43 (43.9)	42 (42.9)		65 (47.8)	62 (45.6)		73 (44.8)	66 (40.5)	
140 kVp, n (%)	3 (3.1)	2 (2.0)		4 (2.9)	3 (2.2)		9 (3.1)	4 (2.5)	
CAD-RADS category			0.407			0.866			0.065
CAD-RADS 0, n (%)	21 (21.4)	14 (14.3)		32 (23.5)	36 (26.5)		49 (30.1)	49 (30.1)	
CAD-RADS 1, n (%)	8 (8.2)	6 (6.1)		8 (5.9)	10 (7.4)		6 (3.7)	15 (9.2)	
CAD-RADS 2, n (%)	34 (34.7)	43 (43.9)		59 (43.4)	52 (38.2)		57 (35.0)	64 (39.3)	
CAD-RADS 3, n (%)	28 (28.6)	28 (28.6)		26 (19.1)	30 (22.1)		35 (21.5)	28 (17.2)	
CAD-RADS 4, n (%)	7 (7.1)	5 (5.1)		8 (5.9)	5 (3.7)		15 (9.2)	5 (3.1)	
CAD-RADS 5, n (%)	0 (0.0)	2 (2.0)		3 (2.2)	3 (2.2)		1 (0.6)	2 (1.2)	
LAD-PCAT (HU)	− 74.83 ± 7.82	− 77.15 ± 8.07	0.067	− 77.21 ± 7.01	− 77.25 ± 7.86	0.996	− 79.02 ± 7.99	− 77.73 ± 7.75	0.185
LCX-PCAT (HU)	− 68.75 ± 7.59	− 71.93 ± 7.25	0.008	− 71.09 ± 6.55	− 71.14 ± 7.40	0.958	− 71.73 ± 8.39	− 71.70 ± 7.57	0.972
RCA-PCAT (HU)	− 74.37 ± 8.44	− 77.20 ± 7.42	0.026	− 76.91 ± 7.98	− 77.07 ± 7.96	0.881	− 76.38 ± 8.91	− 77.33 ± 7.28	0.340

Data are presented as means ± standard deviations or the median (25th and 75th percentile), with the interquartile range in parentheses or number (%)

*T2DM* type 2 diabetes mellitus, *GC* glycemic control, *CAD-RADS* coronary artery disease-reporting and data system, *PCAT* Pericoronary adipose tissue, *LAD* left anterior descending artery, *LCX* left circumflex artery, RCA right coronary artery

The baseline data for T2DM patients divided into three groups based on GC statuses is shown in Additional file 1: Table S1. The results showed that there were significant differences in LAD-PCAT among the three groups from the baseline data (*p* = 0.008).

### Comparison of clinical characteristics and CT parameters in GLDIS T2DM patients and non-GLDIS T2DM patients

**Fig. 4:**
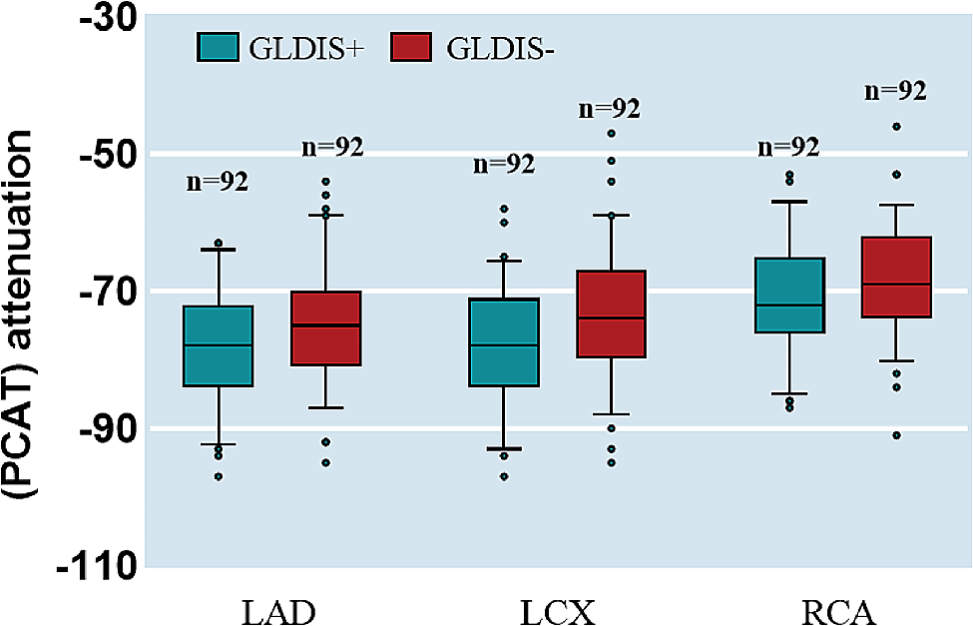
PCAT attenuation in three main coronary arteries stratified by GLDIS. *GLDIS* glucose-lowering drug interventions, *PCAT* Pericoronary adipose tissue, *LAD* left anterior descending artery, *LCX* left circumflex artery, *RCA* right coronary artery

For T2DM patients, the presence or absence of GLDIS made a significant difference in RCA-PCAT, LAD-PCAT, and LCX-PCAT (GLDIS T2DM patients vs. non-GLDIS T2DM patients, LAD: − 75.29 ± 8.33 vs. − 78.29 ± 8.17, *p* = 0.002 ;LCX: − 68.74 ± 7.97 vs. − 72.09 ± 7.93, *p* = 0.001;RCA: − 73.66 ± 9.10 vs. − 77.67 ± 8.53, *p* < 0.001) (Additional file 1: Table S2 and Fig. S1). This significant difference remained after conducting PSM to adjust for cardiovascular risk factors (age, sex, body mass index, dyslipidemia, hypertension and smoking) and statin use rates ( *p*<0.05 for all, Table 4; Fig. 4 ).

**Table 4:** Comparison of clinical characteristics and CT parameters in GLDIS T2DM patients and non-GLDIS T2DM patients after propensity matching

Variables	GLDIS T2DM patients	Non-GLDIS T2DM patients	*P*	
*n*	92	92		
Age (years)	62.08 ± 11.17	62.58 ± 9.01	0.739	
Male sex, n (%)	45 (48.9)	45 (48.9)	1.000	
Body mass index (kg/m2)	25.01 ± 3.88	24.39 ± 3.24	0.344	
Smoking, n (%)	19 (20.7)	18 (19.6)	0.854	
Hypertension, n (%)	59 (64.1)	56 (60.9)	0.648	
Dyslipidemia, n (%)	20 (23.0)	28 (32.9)	0.146	
Statin, n (%)	39 (42.4)	31 (33.7)	0.224	
Tube voltage of CT acquisition			0.499	
100 kVp, n (%)	51 (55.4)	43 (46.7)		
120 kVp, n (%)	36 (39.1)	43 (46.7)		
140 kVp, n (%)	5 (5.4)	6 (6.5)		
LAD-PCAT (HU)	− 78.11 ± 8.01	− 75.04 ± 8.26	0.022	
LCX-PCAT (HU)	− 71.10 ± 8.13	− 68.31 ± 7.90	0.037	
RCA-PCAT (HU)	− 78.17 ± 8.64	− 73.35 ± 9.32	0.001	

Data are presented as means ± standard deviations or the median (25th and 75th percentile), with the interquartile range in parentheses or number (%)

*T2DM* type 2 diabetes mellitus, *GLDIS* glucose-lowering drug interventions, *PCAT* Pericoronary adipose tissue, *LAD* left anterior descending artery, *LCX* left circumflex artery, *RCA* right coronary artery

Subgroup analysis of PCAT attenuation in GLDIS T2DM patients and non-T2DM patients, as well as non-GLDIS T2DM patients and non-T2DM patients, is presented in Additional file 1: Table S3-S4. LCX-PCAT and RCA-PCAT were significantly higher in non-GLDIS T2DM patients compared with non-T2DM patients, while this difference was not observed between GLDIS T2DM patients and non-T2DM patients. The result remains the same even after PSM.

### Results from stepwise regression analysis

Multivariate stepwise linear regression analysis selected five significant factors associated with the attenuation of LAD-PCAT, LCX-PCAT, and RCA-PCAT. Metformin, acarbose and duration of diabetes were found to have a significant impact on LAD-PCAT (metformin : *β* coefficient = − 2.476, *p*=0.021; acarbose : *β* coefficient = − 1.841, *p* = 0.031; Duration of diabetes: *β* coefficient = 0.23, *p*<0.001). Additionally, Tube voltage had a significant impact on all three dependent variables (LAD-PCAT, LCX-PCAT, RCA-PCAT). The independent variables that significantly affected LCX-PCAT and RCA-PCAT are presented in Table 5.

**Table 5:** Multivariate stepwise linear regression for the association between factors and PCAT attenuation

	LAD-PCAT		LCX-PCAT		RCA-PCAT	
	*β* coefficient (95% CI)	*P*	*β* coefficient (95% CI)	*P*	*β* coefficient (95% CI)	*P*
Sex			1.954 (0.399–3.509)	0.014	2.54 (0.808–4.271)	0.004
Duration of T2DM	0.230 (0.105–0.355)	< 0.001	0.114 (0.001–0.227)	0.048		
Metformin	− 2.476 (− 5.101 – − 1.852)	0.021			− 1.89 (− 3.637 – − 0.143)	0.034
Acarbose	− 1.841 (− 3.508 – − 0.174)	0.031				
Tube voltage of CT acquisition	0.351 (0.282–0.42)	< 0.001	0.352 (0.282–0.422)	< 0.001	0.327 (0.25–0.403)	< 0.001

*PCAT* Pericoronary adipose tissue, *LAD* left anterior descending artery, *LCX* left circumflex artery, *RCA* right coronary artery, *CI* confidence interval

## Discussion

To our knowledge, this is a multi-center study with a large sample size, the first to explore more comprehensively the relationship between coronary inflammation based on PCAT attenuation and diabetes management (including GC and GLDIS) in T2DM patients. Firstly, our study revealed that, after adjusting for confounding factors, the RCA-PCAT in poor GC-T2DM patients was significantly higher compared with non-T2DM patients. Secondly, RCA-PCAT, LAD-PCAT, and LCX-PCAT were significantly higher in non-GLDIS T2DM patients than GLDIS T2DM patients. Thirdly, in the multivariate stepwise linear regression, metformin and acarbose were both significantly associated with lower LAD-PCAT. The results indicated inadequate diabetes management, including poor GC and lack of GLDIS, may aggravate coronary inflammation.

This research further substantiates earlier findings of the relationship between T2DM and coronary artery inflammation. A prior study by Yu et al. [18] showed diabetic patients had elevated levels of coronary inflammation compared to non-diabetic populations. Furthermore, a matched case-control study utilizing 18 F-FDG-PET/CT demonstrated type 1 diabetes individuals had increased vascular wall inflammation, correlating with inflammatory blood proteins [29]. Consistently, our results indicated that poor GC-T2DM patients exhibited significantly higher RCA-PCAT versus non-T2DM patients after PSM.

Additionally, this study revealed the potential importance of GLDIS for improving coronary inflammation in T2DM patients. As a new non-invasive technique based on CCTA imaging to directly detect coronary artery inflammation, PCAT attenuation was markedly higher in non-GLDIS T2DM patients compared with GLDIS T2DM patients. This result indicates that non-GLDIS T2DM patients have higher coronary inflammation. Specifically, in the multivariate stepwise linear regression, metformin and acarbose use were significantly associated with lower LAD-PCAT attenuation, indicating targeted medications could play a key role in effective T2DM management to reduce coronary inflammation. Metformin, a first-line medication for T2DM, offers benefits that go beyond GC. It also provides additional cardiovascular protection for T2DM patients. Several studies have shown that its potential to reduce inflammation within blood vessels and improve endothelial function, which can contribute to a lower risk of cardiovascular events. This anti-inflammatory effect may be related to metformin’s impact on certain inflammatory mediators, such as C-reactive protein and interleukin-6, which are known risk factors for cardiovascular diseases [30]. These markers are known risk factors for cardiovascular diseases. Additionally, metformin has been reported to inhibit proinflammatory responses and the activation of Nuclear Factor-kappa B (NF-κB) in human vascular wall cells, which further supports its role in cardiovascular protection [31]. Furthermore, a meta-analysis on the effects of metformin treatment indicated that early therapy with metformin might ameliorate chronic inflammation, as evidenced by reductions in serum levels of CRP [32]. Interestingly, research by Sardu et al. [33] also supports metformin’s potential for alleviating coronary inflammation - non-metformin users had more adipose inflammation, higher leptin/adiponectin ratios, and more cardiovascular events than metformin users, which further supported our results. Our study suggested that metformin use was significantly associated with lower LAD-PCAT attenuation.

Similarly, acarbose (a carbohydrate absorption inhibitor) reduces postprandial hyperglycemia and glucose fluctuations in T2DM patients, thereby mitigating inflammatory responses linked to glycemic variability [28, 34, 35]. These inferences still need to be further verified in the future.

Furthermore, diabetes, a persistent chronic inflammatory illness, is intricately tied to alterations in cardiac structure and function, like diminished left ventricular performance and cardiac enlargement [36]. Elevated blood glucose spurs excessive protein accumulation in the myocardium and rising glycation end products (AGEs) [37]. These stimulate inflammatory signaling and apoptosis pathways in endothelial cells, heightening oxidative stress and inflammation. This exacerbates myocardial cell damage, impacting cardiac structure and function. However, the linkage between heart function and coronary artery inflammation remains sparsely explored, underscoring the need for more extensive investigation in this domain.

This study had several limitations that may affect the interpretation of our results and conclusions. First, the cross-sectional design used in this study means that we cannot determine the causal relationship between diabetes management and coronary artery inflammation, and we also cannot completely rule out the possibility of selection bias. In addition, the study data came from only two tertiary hospitals’ patient populations, which may limit the applicability of our findings to the broader population of patients with type 2 diabetes. Therefore, future research needs to be conducted across multiple centers and cover larger sample sizes to enhance the generalizability and reliability of the conclusions. Second, although we used propensity score matching to adjust for known confounding factors and sample size analysis to ensure the reliability and statistical power of the study, we were unable to consider all potential confounding variables such as dietary habits, physical activity levels, and complications. These unmeasured factors may affect the relationship between diabetes management and coronary artery inflammation. Therefore, future research should include these potential confounding factors and use more comprehensive data collection to reduce their interference with the study results. Third, this study failed to provide detailed information on the duration and dosage of GLDIS, limiting our ability to understand how these factors affect coronary artery inflammation. This highlights the necessity for future research to collect and analyze detailed information such as patients’ medication adherence, specific dosages, and treatment durations to assess the effects of GLDIS more accurately on coronary artery health. In summary, we recognize that selection bias, unmeasured confounding factors, and the lack of detailed GLDIS dosage and usage time information may affect our study results. Future research should adopt multi-center, prospective longitudinal cohort study designs and include more potential confounding factors such as dietary habits, physical activity levels, and complications. Through more rigorous study designs and comprehensive data collection methods, we can more accurately assess the correlation between cardiovascular events and PCAT attenuation and GLDIS, further validating the efficacy and reliability of the study. This can provide more convincing evidence and a deeper understanding of the connections between diabetes management, coronary artery inflammation, and cardiovascular events.

## Conclusions

Our research indicated that RCA-PCAT attenuation was significantly higher in poor GC-T2DM compared with non-T2DM. Additionally, PCAT attenuation was significantly lower in GLDIS T2DM patients versus non-GLDIS T2DM patients. Our research reveals a preliminary signal that inadequate diabetes management, including poor GC and lack of GLDIS, may be associated with increased coronary artery inflammation in T2DM patients, as indicated by PCAT attenuation on CCTA. These insights could assist healthcare providers in identifying T2DM patients at increased cardiovascular risk and developing improved protocols for cardiovascular management, with the potential to reduce subsequent cardiovascular-related mortality.

### Electronic supplementary material

Below is the link to the electronic supplementary material.

**Additional file 1**. Table S1. Comparison of clinical characteristics and CT parameters in diabetic patients according to GC status. Table S2. Comparison of clinical characteristics and CT parameters in GLDIS T2DM patients and non-GLDIS T2DM patients. Table S3. Comparison of clinical characteristics and CT parameters in GLDIS T2DM patients and non-T2DM patients. Table S4. Comparison of clinical characteristics and CT parameters in non-GLDIS T2DM patients and non-T2DM patients. Figure S1. PCAT attenuation in three main coronary arteries stratified by GLDIS.

## Data Availability

No datasets were generated or analysed during the current study.

## Abbreviations

T2DM: Type 2 diabetes mellitus
GLDIS: Glucose-lowering drug interventions
GC: Glycemic control
CAD: Coronary artery disease
CTTA: Coronary Computed tomography angiography
PCAT: Pericoronary adipose tissue
LAD: Left anterior descending artery
LCX: Left circumflex artery
RCA: Right coronary artery
CVD: Cardiovascular disease
BMI: Body mass index
HbA1c: Glycated hemoglobin
LDL: Low-density lipoprotein
HDL: High-density lipoprotein
HU: Hounsfield units
DS: Diameter stenosis
PSM: Propensity score matching
IQR: Interquartile range
SD: Standard deviation
