# Supplementary material for: Coronary inflammation based on pericoronary adipose tissue attenuation in type 2 diabetic mellitus: effect of diabetes management
Source: Cardiovasc Diabetol. 2024 Mar 29;23:108. doi: 10.1186/s12933-024-02199-x (PMC10981289; doi:10.1186/s12933-024-02199-x)
Supplement: Supplementary file 1 — Additional file 1. Table S1. Comparison of clinical characteristics and CT parameters in diabetic patients according to GC status. Table S2. Comparison of clinical characteristics and CT parameters in GLDIS T2DM patients and non-GLDIS T2DM patients. Table S3. Comparison of clinical characteristics and CT parameters in GLDIS T2DM patients and non-T2DM patients. Table S4. Comparison of clinical characteristics and CT parameters in non-GLDIS T2DM patients and non-T2DM patients. Figure S1. PCAT attenuation in three main coronary arteries stratified by GLDIS. [file 12933_2024_2199_MOESM1_ESM.docx]

**Table S1** Comparison of clinical characteristics and CT parameters in diabetic patients according to GC statu

| Variables | | All | Well GC-T2DM | Moderate GC-T2DM | Poor GC-T2DM | P |
| --- | --- | --- | --- | --- | --- | --- |
|  |  |  |  |  |  |  |
| n | | 547 | 209 | 195 | 143 |  |
| Baseline characteristic | |  |  |  |  |  |
|  | Age (years) | 62.35±9.77 | 63.95±10.393 | 62.47±9.283 | 59.87±8.999 | < 0.001 |
|  | Male sex, n (%) | 281 (51.3) | 119 (56.9） | 88 (45.1） | 74 (51.7） | 0.06 |
|  | Body mass index (kg/m2) | 24.22 (22.58,26.29) | 24.22 (22.07,26.23） | 24.49 (22.86,26.22） | 23.77 (22.52,26.44） | 0.309 |
|  | Smoking, n (%) | 138 (25.2) | 49 (23.4） | 45 (23.1） | 44 (30.8） | 0.206 |
|  | Hypertension, n (%) | 353 (64.4) | 147 (70.3） | 123 (63.1） | 82 (57.3） | 0.04 |
|  | Dyslipidaemia, n (%) | 352 (64.4) | 76 (36.4） | 88 (45.1） | 78 (54.5） | 0.003 |
| Diabetes features | |  |  |  |  |  |
|  | TyG | 9.16 (8.70,9.75） | 8.95 (8.51,9.35） | 9.18 (8.76,9.72） | 9.63 (9.01,10.12） | < 0.001 |
|  | Duration of diabetes (years) |  |  |  |  | 0.006 |
|  | <5 (%) | 200 (42.9） | 103 (49.3） | 71 (36.4） | 56 (39.2） |  |
|  | 5–10 (%) | 84 (18.0） | 47 (22.5） | 38 (19.5） | 24 (16.8） |  |
|  | >10 (%) | 182 (39.1） | 59 (28.2） | 86 (44.1） | 63 (44.1） |  |
|  | Complications of diabetes (%) | 282 (51.6） | 88 (42.1） | 111 (56.9） | 83 (58） | 0.002 |
|  | Newly diagnosed T2DM (%) | 83 (15.4） | 40 (19.1） | 21 (10.8） | 22 (15.4） | 0.064 |
|  | Antihypertensive drugs (%) | 223 (40.8) | 108 (51.7） | 80 (41.0） | 43 (30.1） | < 0.001 |
| Medications treatment | |  |  |  |  |  |
|  | Statin, n (%) | 345 (63.0) | 121 (57.9） | 126 (64.6） | 98 (68.5） | 0.109 |
|  | Insulin (%) | 110 (20.1) | 17 (8.1） | 49 (25.1） | 44 (30.8） | < 0.001 |
|  | Metformin (%) | 223 (40.7） | 82 (39.0） | 83 (42.6） | 58 (40.6） | 0.792 |
|  | DDP-4 inhibitors,n (%) | 189 (34.5) | 54 (26.0） | 70 (35.9） | 65 (45.5） | 0.001 |
|  | Sulfonylurea, n (%) | 69 (12.6) | 27 (13.0） | 25 (12.8） | 17 (11.9） | 0.951 |
|  | Thiazolidinedione drugs (%) | 62 (11.3) | 18 (8.7） | 24 (12.3） | 20 (14） | 0.264 |
| Laboratory findings | |  |  |  |  |  |
|  | Fast glucose (mmol/L) | 7.84 (6.35,10.48) | 6.60 (5.71,7.54） | 8.18 (6.79,9.94） | 11.51 (8.89,14.25） | < 0.001 |
|  | HbA1c (%) | 7.70 (6.60,9.20) | 6.30 (6,6.8） | 7.90 (7.40,8.40） | 10.7 (9.80,11.75） | < 0.001 |
|  | HDL-cholesterol (mmol/L) | 1.13 (0.97,1.37) | 1.19 (1.00,1.42） | 1.10 (0.97,1.37） | 1.07 (0.90,1.31） | 0.011 |
|  | LDL-cholesterol (mmol/L) | 2.62 (2.07,3.34) | 2.41 (1.58,2.99） | 2.70 (2.11,3.53） | 2.90±0.94 | 0.001 |
|  | Total cholesterol (mmol/L) | 4.58±1.13 | 4.26 (3.63,4.93） | 4.63±1.10 | 4.83±1.20 | < 0.001 |
|  | Triglyceride (mmol/L) | 1.55 (0.99,2.39) | 1.47 (0.96,2.02） | 1.55 (1.00,2.49） | 1.64 (1.03,2.75） | 0.067 |
|  | No diabetic treatment | 108 (19.7） | 51 (24.4） | 28 (14.4） | 29 (20.3） | 0.04 |
| CCTA findings | |  |  |  |  |  |
|  | Tube voltage of CT acquisition | |  |  |  | 0.589 |
|  | 100 kVp, n (%) | 297 (54.2) | 117 (56） | 100 (51.3） | 80 (55.9） |  |
|  | 120 kVp, n (%) | 229 (41.8) | 87 (41.6） | 85 (43.6） | 57 (39.9） |  |
|  | 140 kVp, n (%) | 21 (3.8) | 5 (2.4） | 10 (5.1） | 6 (4.2） |  |
|  | DS | 192 (35.1) | 67 (32.1） | 66 (33.8） | 59 (41.3） | 0.186 |
|  | LAD-PCATA (HU) | − 77.64±8.27 | − 78.78±8.012 | − 77.93±7.676 | − 75.65±9.151 | 0.008 |
|  | LCX-PCATA (HU) | − 71.40±8.04 | − 72.07±8.249 | − 71.71±7.117 | − 70.02±8.861 | 0.103 |
|  | RCA-PCATA (HU) | − 76.84±8.80 | − 76.76±8.805 | − 77.37±8.311 | − 76.13±9.447 | 0.523 |

Data are presented as means ± standard deviations or the median, with the interquartile range in parentheses or number (%)

*T2DM* type 2 diabetes mellitus, *GC* glycemic control, *HbA1c* glycated haemoglobin, *HDL* high-density lipoprotein, *LDL* low-density lipoprotein, *DS* diameter stenosis, *PCAT* Pericoronary adipose tissue, *LAD* left anterior descending artery, *LCX* left circumflex artery, RCA right coronary artery

**Table S2** Comparison of clinical characteristics and CT parameters in GLDIS T2DM and non-GLDIS T2DM patients patients

| Variables | GLDIS T2DM patients | Non-GLDIS T2DM patients | P |  |
| --- | --- | --- | --- | --- |
| n | 439 | 108 |  |  |
| Age (years) | 62.49±9.88 | 61.72±9.22 | 0.482 |  |
| Male sex, n (%) | 226 (51.5) | 55 (50.9) | 0.918 |  |
| Body mass index (kg/m2) | 24.74±3.56 | 24.39±3.24 | 0.562 |  |
| The duration of diabetes (years) | 9.16±6.48 | 1.68±3.72 | <0.001 |  |
| Smoking, n (%) | 115 (26.2) | 23 (21.3) | 0.294 |  |
| Hypertension, n (%) | 284 (64.7) | 68 (63.0) | 0.737 |  |
| Dyslipidaemia, n (%) | 187 (42.6) | 55 (50.9) | 0.118 |  |
| Statin, n (%) | 307 (69.9) | 38 (35.2) | <0.001 |  |
| Fast glucose (mmol/L) | 8.02 (6.47,11.04) | 7.28 (5.97,9.28) | 0.050 |  |
| HbA1c (%) | 7.80 (6.60,9.20) | 7.30 (6.30,9.80) | 0.140 |  |
| HDL-cholesterol (mmol/L) | 1.13 (0.97,1.37) | 1.11 (0.98,1.37) | 0.700 |  |
| LDL-cholesterol (mmol/L) | 2.55 (2.02,3.29) | 3.02±0.95 | <0.001 |  |
| Total cholesterol (mmol/L) | 4.51±1.13 | 4.86±1.12 | 0.006 |  |
| Triglyceride (mmol/L) | 1.51 (0.97,2.3) | 1.71 (1.16,2.44) | 0.090 |  |
| Tube voltage of CT acquisition |  |  | 0.398 |  |
| 100 kvp, n (%) | 245 (55.8) | 53 (49.1) |  |  |
| 120 kvp, n (%) | 177 (40.3) | 49 (45.4) |  |  |
| 140 kVp, n (%) | 17 (3.9) | 6 (5.6) |  |  |
| LAD-PCAT (HU) | − 78.29±8.17 | − 75.29±8.33 | 0.002 |  |
| LCX-PCAT (HU) | − 72.09±7.93 | − 68.74±7.97 | 0.001 |  |
| RCA-PCAT (HU) | − 77.67±8.53 | − 73.66±9.10 | <0.001 |  |

Data are presented as means ± standard deviations or the median (25th and 75th percentile) , with the interquartile range in parentheses or number (%)

*T2DM* type 2 diabetes mellitus, *GLDIS* glucose-lowering drug interventions, *HbA1c* glycated haemoglobin, *PCAT* Pericoronary adipose tissue, *LAD* left anterior descending artery, *LCX* left circumflex artery, RCA right coronary artery, *HDL* high-density lipoprotein, *LDL* low-density lipoprotein

**Table S3** Comparison of clinical characteristics and CT parameters in GLDIS T2DM patients and non-T2DM patients

| Variables | Total Cohorts (n =1234) | |  | Matched Cohorts (n = 542) | |  |
| --- | --- | --- | --- | --- | --- | --- |
|  | GLDIS T2DM patients (n = 439) | Non-T2DM patients (n =795) | P | GLDIS T2DM patients (n = 271) | Non-T2DM patients (n =271) | P |
| Age (years) | 62.49±9.88 | 62.49±10.71 | 0.993 | 62.49±10.23 | 62.77±10.50 | 0.761 |
| Male sex, n (%) | 226 (51.5) | 381 (47.9) | 0.232 | 131 (48.3） | 133 (49.1） | 0.864 |
| Body mass index (kg/m2) | 24.74±3.56 | 23.73 (22.03,26.01) | 0.049 | 24.21 (22.60,26.19) | 24.06 (22.31,26.35) | 0.735 |
| Smoking, n (%) | 115 (26.2) | 147 (18.5) | 0.002 | 64 (23.6) | 54 (19.9） | 0.298 |
| Hypertension, n (%) | 284 (64.7) | 372 (46.9) | < 0.001 | 162 (59.8） | 152 (56.1） | 0.384 |
| Dyslipidemia, n (%) | 187 (42.6) | 280 (35.2) | 0.011 | 116 (42.8） | 119 (43.9） | 0.795 |
| Statin, n (%) | 307 (69.9) | 321 (40.4) | < 0.001 | 170 (62.7) | 175 (64.6) | 0.655 |
| Tube voltage of CT acquisition |  |  | 0.008 |  |  | 0.502 |
| 100 kVp, n (%) | 245 (55.8) | 475 (59.7) |  | 157 (57.9) | 148 (54.6) |  |
| 120 kVp, n (%) | 177 (40.3) | 310 (39.4) |  | 108 (39.9) | 113 (41.7) |  |
| 140 kVp, n (%) | 17 (3.9) | 10 (1.3) |  | 6 (2.2) | 10 (3.7) |  |
| LAD-PCAT (HU) | − 78.29±8.17 | − 76.98±7.93 | 0.028 | − 78.03±7.333 | − 77.29±7.844 | 0.307 |
| LCX-PCAT (HU) | − 72.09±7.93 | − 71.23±7.29 | 0.112 | − 71.55±7.155 | − 71.43±7.708 | 0.869 |
| RCA-PCAT (HU) | − 77.67±8.53 | − 77.08±7.42 | 0.204 | − 77.08±7.95 | − 77.34±7.497 | 0.721 |

Data are presented as means ± standard deviations or the median (25th and 75th percentile) , with the interquartile range in parentheses or number (%)

*T2DM* type 2 diabetes mellitus, *GLDIS* glucose-lowering drug interventions, *PCAT* Pericoronary adipose tissue, *LAD* left anterior descending artery, *LCX* left circumflex artery, RCA right coronary artery

**Table S4** Comparison of clinical characteristics and CT parameters in non-GLDIS T2DM patients and non-T2DM patients

|  | Total Cohorts (n =1234) | |  | Matched Cohorts (n = 960) | |  |
| --- | --- | --- | --- | --- | --- | --- |
|  | Non-GLDIS T2DM patients (n = 108) | Non-T2DM patients (n =795) | P | Non-GLDIS T2DM patients (n = 90) | Non-T2DM patients (n =90) | P |
| Age (years) | 61.72±9.22 | 62.49±10.71 | 0.522 | 62.66±8.97 | 62.63±10.9 | 0.833 |
| Male sex, n (%) | 55 (50.9) | 381 (47.9) | 0.558 | 43 (47.8) | 47 (52.2) | 0.655 |
| Body mass index (kg/m2) | 24.39±3.24 | 23.73 (22.03,26.01) | 0.422 | 24.38±3.26 | 24.76±3.79 | 0.473 |
| Smoking, n (%) | 23 (21.3) | 147 (18.5) | 0.484 | 17 (18.9) | 22 (24.4) | 0.366 |
| Hypertension, n (%) | 68 (63.0) | 372 (46.9) | 0.002 | 54 (60.0) | 58 (64.4) | 0.539 |
| Dyslipidemia, n (%) | 55 (50.9) | 280 (35.2) | 0.002 | 46 (51.1) | 52 (57.8) | 0.369 |
| Statin, n (%) | 38 (35.2) | 321 (40.4) | 0.301 | 37 (41.1) | 43 (47.8) | 0.368 |
| Tube voltage of CT acquisition |  |  | 0.013 |  |  | 0.755 |
| 100 kVp, n (%) | 53 (49.1) | 475 (59.7) |  | 41 (45.6) | 46 (51.1) |  |
| 120 kVp, n (%) | 49 (45.4) | 310 (39.4) |  | 48 (53.3) | 43 (47.8) |  |
| 140 kVp, n (%) | 6 (5.6) | 10 (1.3) |  | 1 (1.1) | 1 (1.1) |  |
| LAD-PCAT (HU) | − 75.29±8.33 | − 76.98±7.93 | 0.071 | − 75.21±8.274 | − 77.45±7.388 | 0.079 |
| LCX-PCAT (HU) | − 68.74±7.97 | − 71.23±7.29 | 0.001 | − 68.37±7.915 | − 71.55±7.611 | 0.014 |
| RCA-PCAT (HU) | − 73.66±9.10 | − 77.08±7.42 | 0.007 | − 73.46±9.343 | − 77.43±7.475 | 0.004 |

Data are presented as means ± standard deviations or the median (25th and 75th percentile) , with the interquartile range in parentheses or number (%)

*T2DM* type 2 diabetes mellitus, *GLDIS* glucose-lowering drug interventions, *PCAT* Pericoronary adipose tissue, *LAD* left anterior descending artery, *LCX* left circumflex artery, *RCA* right coronary artery

**Fig. S1** PCAT attenuation in three main coronary arteries stratified by GLDIS. *GLDIS* glucose-lowering drug interventions, *LAD* left anterior descending artery, *LCX* left circumflex artery, *RCA* right coronary artery
